# Global expression profiling of theophylline response genes in macrophages: evidence of airway anti-inflammatory regulation

**DOI:** 10.1186/1465-9921-6-89

**Published:** 2005-08-08

**Authors:** Pei-Li Yao, Meng-Feng Tsai, Yi-Chen Lin, Chien-Hsun Wang, Wei-Yu Liao, Jeremy JW Chen, Pan-Chyr Yang

**Affiliations:** 1 Department of Internal Medicine, National Taiwan University Hospital, No. 7, Chung-Shan South Rd., Taipei 100, Taiwan; 2 NTU Center for Genomic Medicine, National Taiwan University College of Medicine, Taipei 100, Taiwan; 3 Institutes of Biomedical Sciences and Molecular Biology, National Chung-Hsing University, No. 250, Kuo-Kuang Rd., Taichung 40227, Taiwan

## Abstract

**Background:**

Theophylline has been used widely as a bronchodilator for the treatment of bronchial asthma and has been suggested to modulate immune response. While the importance of macrophages in asthma has been reappraised and emphasized, their significance has not been well investigated. We conducted a genome-wide profiling of the gene expressions of macrophages in response to theophylline.

**Methods:**

Microarray technology was used to profile the gene expression patterns of macrophages modulated by theophylline. Northern blot and real-time quantitative RT-PCR were also used to validate the microarray data, while Western blot and ELISA were used to measure the levels of IL-13 and LTC4.

**Results:**

We identified dozens of genes in macrophages that were dose-dependently down- or up-regulated by theophylline. These included genes related to inflammation, cytokines, signaling transduction, cell adhesion and motility, cell cycle regulators, and metabolism. We observed that IL-13, a central mediator of airway inflammation, was dramatically suppressed by theophylline. Real-time quantitative RT-PCR and ELISA analyses also confirmed these results, without respect to PMA-treated THP-1 cells or isolated human alveolar macrophages. Theophylline, rolipram, etazolate, db-cAMP and forskolin suppressed both IL-13 mRNA expression (~25%, 2.73%, 8.12%, 5.28%, and 18.41%, respectively) and protein secretion (<10% production) in macrophages. These agents also effectively suppressed LTC4 expression.

**Conclusion:**

Our results suggest that the suppression of IL-13 by theophylline may be through cAMP mediation and may decrease LTC4 production. This study supports the role of theophylline as a signal regulator of inflammation, and that down regulation of IL-13 by theophylline may have beneficial effects in inflammatory airway diseases.

## Introduction

Asthma is a highly prevalent health problem worldwide that may cause significant morbidity and mortality [1,2]. The mechanisms of airflow obstruction in asthma are various, including broncho-constriction with the contraction of the airway's smooth muscle, increased secretion of mucus, mucosal edema with vascular leakage, and the infiltration of inflammatory cells [3]. The pathogenesis of asthma and its susceptibility involve a complex interplay of various genetic and environmental factors, which may modulate airway inflammation and the remodeling processes that are not only present even in mild asthma but also govern the appearance and severity of airway hyper-responsiveness [4].

The inflammatory cells involved include the infiltration of airway T cells, T helper cells, mast cells, basophils, eosinophils, and macrophages [5]. Macrophages are the predominant immune effector in the alveolar spaces and airway, and are believed to play a pivotal role in various pulmonary inflammatory disorders [6,7]. Recently, their importance in the pathogenesis of asthma has been reappraised and emphasized [8]. Although their role in asthmatic inflammation is still incompletely understood, it is clear that macrophages may participate in airway inflammation though multiple mechanisms. Furthermore, macrophages have been reported to release lukotriene B4 (LTB4), lukotriene C4 (LTC4), prostaglandin D2 (PGD2), superoxide anion, and lysosomal enzymes in response to immunoglobulin E (Ig E) [5,9,10]. They also produce inflammatory mediators, such as platelet-activating factor, interleukin 1 beta (IL1β), IL-6, IL-8, and tumor necrosis factor- alpha (TNF-α) [11-14]. These mediators may play important roles in producing broncho-constriction or causing inflammatory changes.

Theophylline is a weak and non-selective inhibitor of phosphodiesterase (PDE) in airway smooth muscle cells. In high doses, theophylline may lead to an increase in intracellular cAMP and cGMP, and mediate the relaxation of airway smooth muscles and suppress airway inflammation [15]. In chronic obstructive pulmonary disease (COPD) patients, theophylline can reduce the total number and proportion of neutrophils, the production of interleukin-8, and neutrophil chemotatic responses, further suggesting its anti-inflammatory effects [15,16]. Several studies have also demonstrated that theophylline has a steroid-sparing effect [17,18]. Theophylline inhibits the degranulation and release of mediators, including platelet-activating factor, LTC4, cationic proteins, and superoxide anion, from eosinophils, granulocytes, and alveolar macrophages *in vitro *[19,20]. However, the effects of theophylline on gene expressions in macrophages has not been well studied.

In this study, we analyzed the expression profiles of inflammation-related genes of macrophages in response to theophylline, using a human cDNA microarray [21,22]. We also identified differentially expressed genes in macrophages after incubating with theophylline. Our study confirmed the diverse roles of theophylline as an immune modulator, which may be helpful in improving its use in the treatment of airway inflammatory disorders.

## Methods

### Cell lines, alveolar macrophage isolation, and theophylline treatment

Human monocyte cell line THP-1 (ATCC TIB 202; ATCC, Manassas, VA) was grown with RPMI 1640 media (GIBCO-BRL; Gaithersburg, MD) supplemented with 1.5 g/l Na_2_HCO_3_, 4.5 g/l glucose and 10% FBS (GIBCO-BRL) and then incubated at 37°C with 20% O_2 _and 5% CO_2· _3.2 × 10^-7^M PMA (SIGMA Chemical Co.; St. Louis, MO) was applied to monocyte cultures. After incubating with PMA for 24 hours, monocytes were differentiated into macrophage-like phenotypes. Macrophages were washed three times with RPMI medium containing 10% FBS and incubated for another 24 hours to eliminate the effects of PMA.

Alveolar macrophages were obtained by bronchoalveolar lavage (BAL) during routine bronchoscopic examination with written informed consent from three smoker patients with chronic bronchitis. BAL was performed from the right middle lobe or lingula using three to five successive aliquots of 20 ml of 0.9% sterile NaCl. The BAL fluid was centrifuged at 800 × g for 10 min at 4°C. After two washings, the cells were plated on plastic Petri dishes in serum-free RPMI 1640 media and allowed to adhere for 2 h at 37°C. Non-adherent cells were removed by washings with PBS. Adherent cells contained more than 95% alveolar macrophages [23,24]. The 5 × 10^4 ^cells were plated on 24 well plates with complete RPMI medium. After incubating for 24 hours, theophylline was added to the alveolar macrophages. The study protocol was approved by the National Taiwan University Hospital's Ethics Committee.

The designated concentration of theophylline (0, 2.5, 5, 10, and 20 μg/ml; SIGMA) was added to macrophages (PMA-treated THP-1 cells). The drug treatments covered a proper range of theophylline concentrations corresponding to the clinical plasma therapeutic levels for asthma patients [17,25]. After incubation for 24 hours, the cells were harvested with RNAzol B and followed by microarray experiments.

### Human cDNA microarray analysis

Human EST clones with putative gene names were obtained from the IMAGE consortium libraries through its distributor (Research Genetics, Huntsville, AL). The cDNA microarray with 9,600 PCR-amplified cDNA fragments was prepared by an arraying machine. Five micrograms of mRNAs were labeled with Biotin-16-dUTP during the reverse transcription as described in our previous report [22]. All of the experiments were individually performed in triplicate. The microarray images were scanned, digitized, and analyzed using a flat scanner (PowerLook 3000, UMAX, Taipei, Taiwan) and GenePix 3.0 software (Axon, Union City, CA). The replicates were used to calculate the mean and standard deviation of gene expression and the coefficient of variation (CV) as the measurement of reproducibility. The details of target preparation, hybridization, color development, image analysis, and spot quantification have been described previously [21,22]. (*see *online supplemental data for additional details on the microarray system) (see additional file: 1).

### Northern blotting and real-time quantitative RT-PCR

To confirm the results derived from the microarray, six differentially expressed clones were randomly selected from the cluster analysis and the entire inserts of the clones were individually PCR-amplified to serve as probes for Northern blotting. The amplified cDNA fragments were labeled with digoxigenin-11-dUTP by random primed labeling as our previous report [21]. To correct the quantity of RNA loading, the signals were normalized with the mRNA expression level of GAPDH in the same blot.

Due to the limitations of mRNA extraction from non-proliferated macrophages and low expression levels of some genes, we employed real-time quantitative RT-PCR (RTQ-RT-PCR) with SYBR Green detection to confirm the results derived from the microarray. There were eight differentially expressed clones randomly selected from the cluster analysis for RTQ-RT-PCR analyses. The TATA box binding protein (*TBP*) was used as an internal control. The primers were shown in Table 1 and detailed procedures have been described previously [22]. All of the experiments were performed in triplicate.

**Table 1:** Oligonucleotides for real-time quantitative RT-PCR

mRNA targets	Oligonucleotides (5'→3') ^a^	Product size (bp)
IL-5	F180: ATAGCCAATGAGACTCTGAGGATTC	89
	R268: AGTGTGCCTATTCCCTGAAAGAT	
IL-13	F155: TGAGGAGCTGGTCAACATCA	76
	R230: CAGGTTGATGCTCCATACCAT	
IL-18	F211: GCTGAACCAGTAGAAGACAATTGC	94
	R304: CCAGGTTTCATCATCTTCAGCTA	
IL-13Rα1	F495: GGAATACCAGTCCCGACACTAACT	93
	R587: GGCCTTCTCTAAAGATGTTTTCACA	
IL-13Rα2	F44: GGCTATTTGAAGTCGCCATAACC	78
	R121: AGATTTAAAACCTTGATATTGCCTCTCT	
TNF-α	F414: CTCGAACCCCGAGTGACAA	64
	R477: AGCTGCCCCTCAGCTTGA	
VEGF-a	F1200: AACACACACTCGCGTTGCAA	69
	R1268: CGGCTTGTCACATCTGCAAGT	
VEGF-c	F1193: AGATGCCTGGCTCAGGAAGA	74
	R1266: ATGTCATGGAATCCATCTGTTGAGT	
GM-CSF	F119: GCCCTGGGAGCATGTGAA	78
	R196: TTCATCTCAGCAGCAGTGTCTCTA	
TBP	F852: CACGAACCACGGCACTGATT	89
	R940: TTTTCTTGCTGCCAGTCTGGAC	

^a ^F and R indicate forward and reverse primers, respectively. Numbers indicate the mRNA sequence position.

### Western blotting analysis and ELISA

The details of nuclear extract preparation and Western blot analysis have been described previously [26]. IL-13 was detected using a 1:500 dilution of mouse monoclonal anti-IL-13 primary antibody, a 1:1000 dilution of HRP-conjugated anti-mouse IgG secondary antibody (Santa Cruz Biotech, Santa Cruz, CA), and the Western blotting luminol reagent (Santa Cruz Biotech) as detection reagent. α-tubulin, used as the control for gel loading, was detected using mouse monoclonal anti-α-tubulin primary antibody (Santa Cruz Biotech). In addition, the cultured medium was collected and centrifuged to remove cellular debris, and the supernatants were frozen at -80°C until assayed by ELISA (R&D System Inc., Minneapolis, MN, USA). IL-13 concentrations were determined by comparison to recombinant standards that run parallel with each batch of assays. Each sample was determined in duplicate. The sensitivity of this ELISA was at < 32 pg/ml.

### Statistical analysis

All of the experiments were performed in triplicate and analyzed by ANOVA (Excel, Microsoft; Taipei, Taiwan). A *P *value < 0.05 was considered statistically significant. In an attempt to reduce variations arising from experimental results of different microarrays, the intensity values of spots from each microarray were re-scaled using a global-scale method. Detailed procedures have been described previously [21,22]. Where appropriate, the data are presented as the mean ± standard deviation. (*see *online supplement for additional details on the microarray data analysis) (see additional file: 1).

## Results

### Microarray analysis

Biotin-labeled probes deriving from mRNAs of macrophages (PMA-treated THP-1 cells) stimulated with different concentrations of theophylline were hybridized to microarrays with 9,600 putative genes to profile the gene expression patterns. The CV was 5.26% and the Pearson correlation coefficient of overall reproducibility for large-scale analyses was 0.98. The results of microarray analyses indicated that 2,724 out of 9,600 EST clones were identified, according to at least one dosage point, whose expression level is larger than the background (> 3,000 intensity units).

Among these, 341 genes displayed more than a 2-fold expression change across all five study-included dosages in theophylline treatment. 75 genes were randomly selected and sequenced retrospectively after differential expressions were found, to assure that they indeed represented the true transcript. 45 genes were up-regulated and 30 genes were down-regulated by theophylline in macrophages (PMA-treated THP-1 cells). A full list of genes and data related to treatment with theophylline were posted at our Web site. . In addition, the gene lists of suppressed and enhanced expression were shown in the online data supplement as Tables 1 and 2 (see additional file: 1).

**Figure 1:**
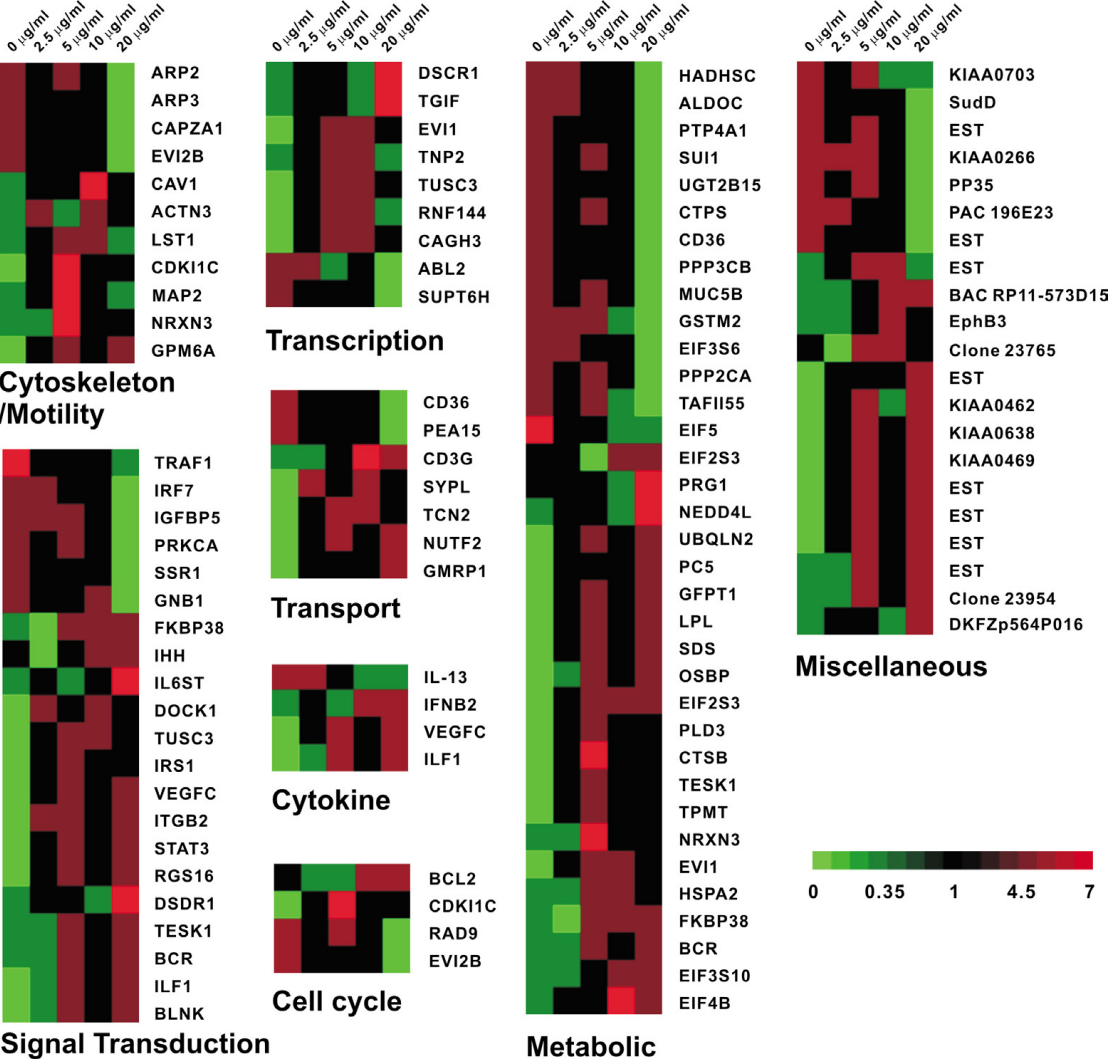
Hierarchical clustering of the gene expression profile in macrophages with or without theophylline. 75 differentially expressed genes dose-dependently down- or up-regulated by theophylline were identified and further grouped into 8 categories. Relative expression levels of these genes are color-coded.

These selected genes were grouped into eight categories by their putative functions on the basis of literature reports (Figure 1). The categories included: (1) cytoskeleton and motility related genes (n = 11), such as *caveolin-1 *and *actin-related protein 3*; (2) signal transduction related genes (n = 21), such as *testis-specific kinase 1 *and *IL-6 signal transducer*, (3) transcription regulators (n = 9), such as *transforming growth β-Induced factor and Down syndrome critical region protein 1*; (4) transport regulators (n = 7), such as *CD36 *and *transcobalamin II*; (5) cytokines (n = 4), such as *IL-13 *and *vascular endothelial growth factor (VEGF)-C*; (6) cell cycle regulators (n = 4), such as *cyclin-dependent kinase inhibitor 1C *and *ecotropic viral integration site 2B*; (7) metabolism related genes (n = 35), such as *platelet proteoglycan 1 *and *eukaryotic translation initiation factor 2, subunit 3*; and (8) miscellaneous genes (unknown) (n = 21), such as KIAA0703 gene and KIAA0266. We found that 51% of affected genes were related to signal transduction or metabolism. Genes with multiple roles were also included in more than one category.

### Northern blotting and RTQ-RT-PCR

**Figure 2:**
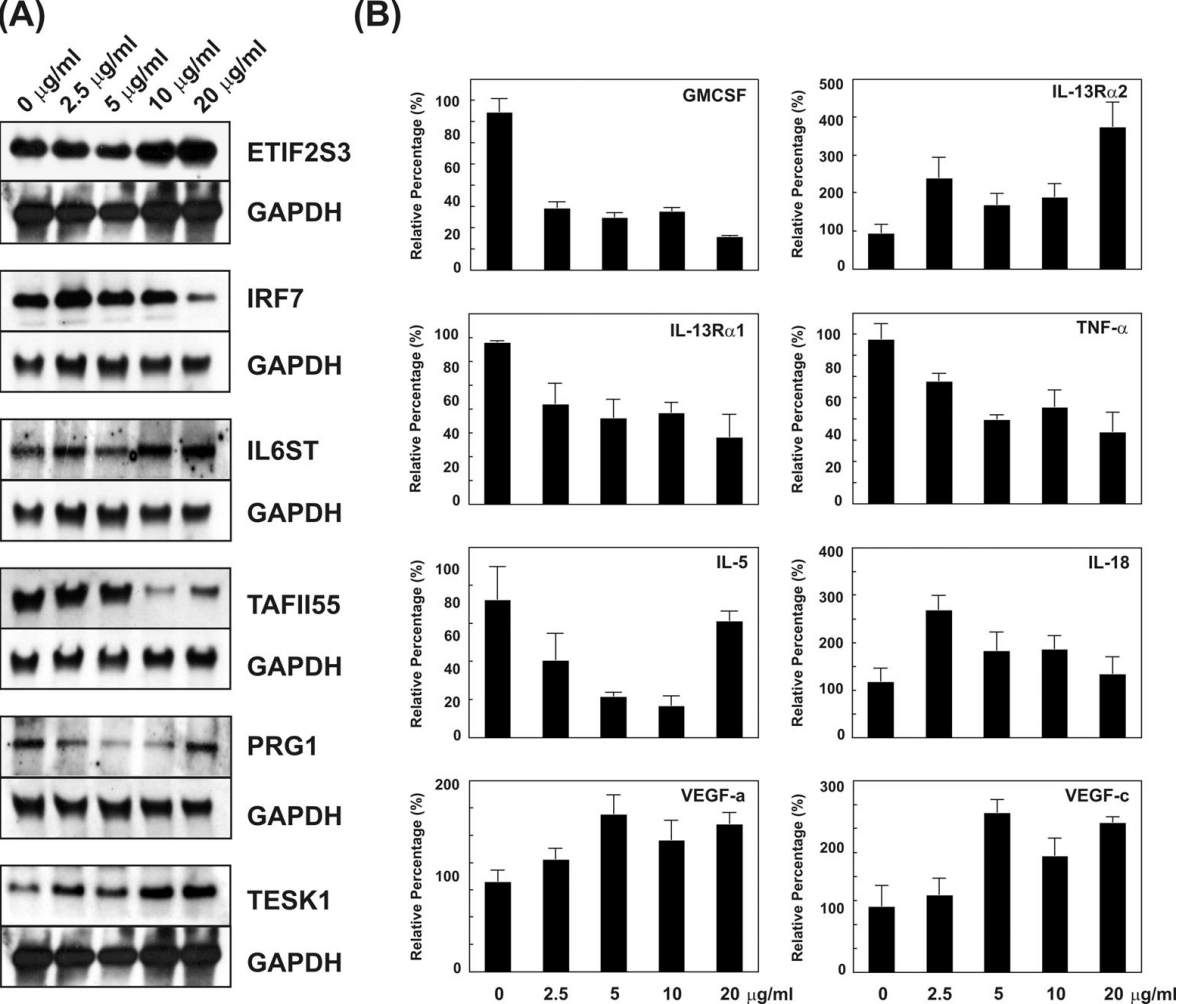
Northern blot and real-time quantitative RT-PCR analyses of differentially expressed genes. (A) Northern blot analysis of six randomly selected genes in macrophages. (B) Real-time quantitative RT-PCR analysis of eight cytokine genes. The relative amount of each cDNA level against to TBP cDNA was measured and defined by an arbitrary unit. (10 μg/ml of theophylline treatment approximately corresponds to the clinical plasma level.)

To substantiate the results of the microarray studies, Northern blot analysis and RTQ-RT-PCR were performed. Six gene expressions that showed more than a 2-fold change, including *ETIF2S3*, *IRF7*, *IL6ST*, *TAFII55*, *PRG1 *and *TESK1*, were randomly selected and evaluated. Figure 2A shows that the results of Northern blot analyses were consistent with of the microarray studies. *GAPDH *was used as an internal control. The other eight genes selected from microarray analysis were also confirmed by RTQ-RT-PCR, including *GMCSF*, *TNF-α*, *IL-13 Rα1*, *IL-13 Rα2*, *IL-5*, *IL-18*, *VEGF-a*, and *VEGF-c *(Figure 2B). The *IRF7*, *TAFII55*, *PRG1*, *GMCSF*, *TNF-α*, *IL-13 Rα1*, *IL-5*, and *IL-18 *genes were suppressed by theophylline, whereas *ETIF2S3*, *IL6ST*, *TESK1*, *IL-13 Rα2*, *VEGF-a*, and *VEGF-c *were stimulated.

### Theophylline down-regulates IL-13 expression

**Figure 3:**
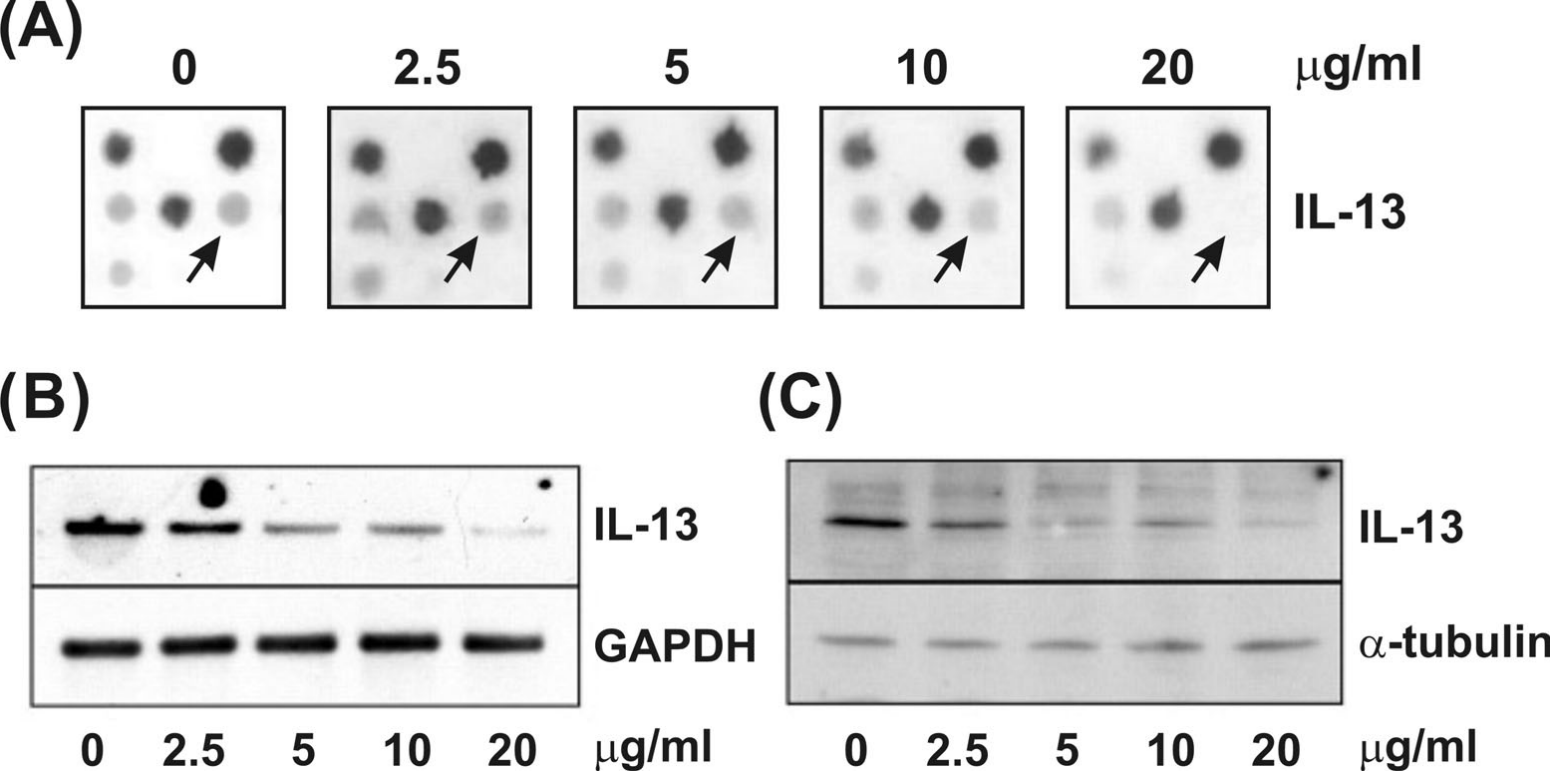
IL-13 expression in macrophages was suppressed by theophylline in a dose-dependent manner. (A) Close-up view of microarray digital image of IL-13 expression. (B) Northern blot analysis of IL-13 mRNA expression in macrophages. GAPDH was used as an internal control. (C) Western blot analysis revealed that IL-13 protein level in macrophages was decreased by theophylline. α-tubulin was used as the loading control. 10 μg/ml of theophylline treatment approximately corresponds to the clinical plasma level.

Microarray analysis revealed that *IL-13 *expression was dose-dependently suppressed by theophylline. Figure 3A revealed a collection of cropped microarray images (3 × 3 spots) showing gene expression patterns of *IL-13 *in macrophages (PMA-treated THP-1 cells) treated with theophylline. Northern and Western blot analyses also showed a similar suppression of IL-13 production (Figure 3B and 3C). The concentration of 10 μg/ml of theophylline approximately corresponds to the clinical plasma therapeutic level.

**Figure 4:**
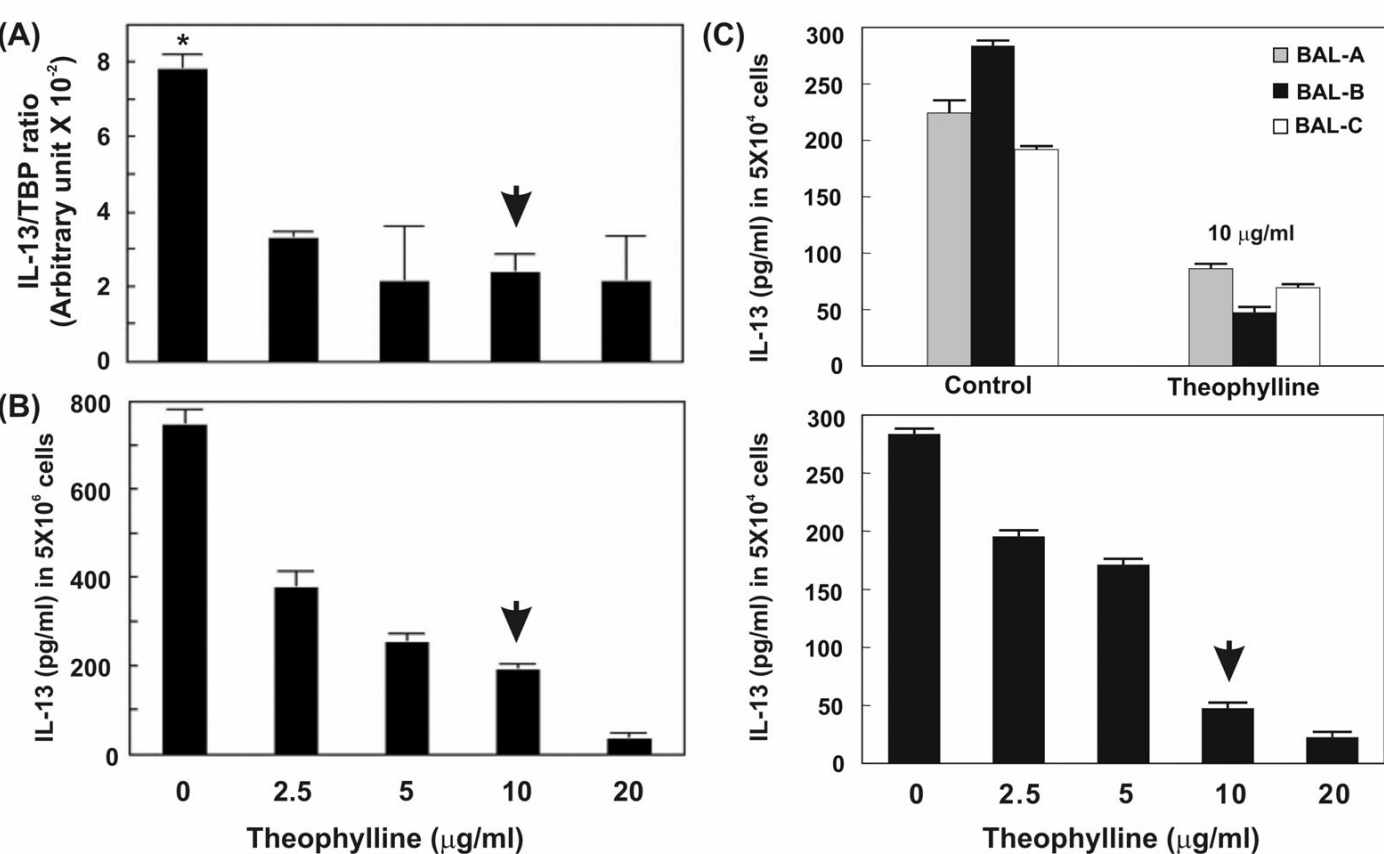
Effects of theophylline on IL-13 expression and protein secretion in macrophages. (A) IL-13 mRNA level was measured by RTQ-RT-PCR, and significantly decreased after treating with theophylline (down to less than 45% compared with control. *α = 0.05, *p *= 0.0079). (B) IL-13 protein secretion, by ELISA analysis, was also reduced in macrophages treated with theophylline. The trend was similar to that for the mRNA (down to less than 55% compared with control. *α = 0.05, *p *= 0.0075). (C) The IL-13 protein secretion in alveolar macrophages isolated from three patients (BAL-A, BAL-B, and BAL-C) with chronic bronchitis was also reduced when treated with 10 μg/ml theophylline (α = 0.05, *p *= 0.043; upper panel). The BAL-B specimens were treated with difference concentration of theophylline (0, 2.5, 5, 10, 20 μg/ml, respectively). IL-13 protein secretion was decreased in a dose-dependent manner with theophylline (lower panel). Arrow indicates the concentration of theophylline treatment corresponding to the clinical plasma levels (10 mg/L).

IL-13 mRNA expression in macrophages (PMA-treated THP-1 cells) with different dosages of theophylline treatment was measured by RTQ-RT-PCR, and results showed a significant suppression compared with the control (α = 0.05, *p *= 0.0079) (Figure 4A). ELISA showed that IL-13 protein secretion was also reduced in a dose-dependent manner (50.23%, 32.43%, 24.93%, and 5.33%, respectively, of the level seen in the absence of theophylline) (Figure 4B).

In this study, we also evaluated IL-13 expression in human alveolar macrophages using ELISA. Results showed that IL-13 protein secretion was reduced in alveolar macrophages when treated by 10 μg/ml theophylline. The amounts of IL-13 protein in those without theophilline treatment specimens BAL-A, BAL-B and BAL-C are 224, 283 and 191 pg/ml, respectively. In contrast, there are 86, 47, and 69 pg/ml of IL-13 in the respective theophylline treatment specimens. In alveolar macrophages from smoker patients with chronic bronchitis, IL-13 protein secretion was decreased in a dose-dependent manner with theophylline (Figure 4C).

### cAMP-dependent pathways in the down-regulation of IL-13 expression

**Figure 5:**
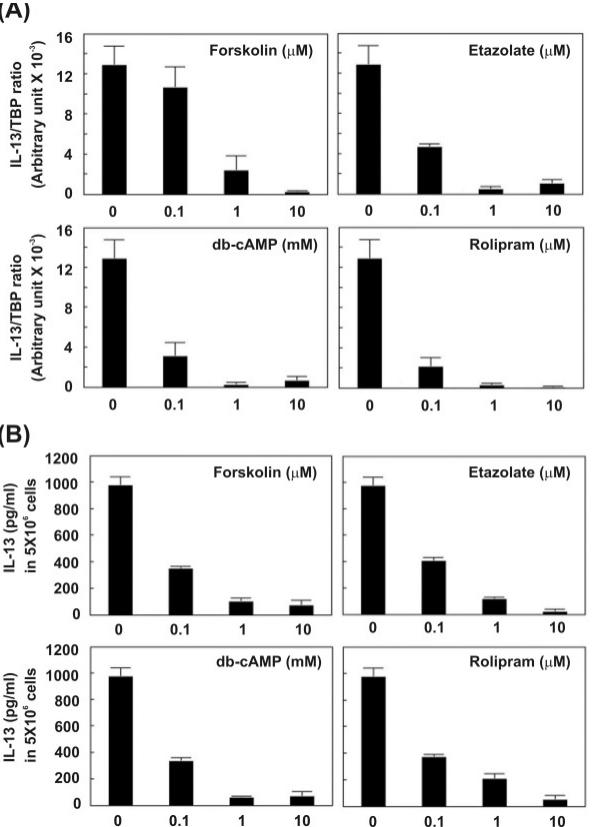
Suppression of IL-13 expression in macrophages by PDE type IV inhibitors and cAMP-elevating agents. Two PDE type IV inhibitors, etazolate and rolipram, and two cAMP-elevating agents, forskolin and db-cAMP (dibutyryl-cAMP), were added to macrophage separately for 24 hours. The cells were harvested to extract RNA for RTQ-RT-PCR, and the cultured medium were used to carry out ELISA. (A) RTQ-RT-PCR analysis showed a decrease of IL-13 mRNA in a dose-dependent manner after treating with four drugs (α = 0.05, *p *= 0.0009). (B) The results of ELISA also revealed that IL-13 protein secretion was reduced after treatment with four drugs (α = 0.05, *p *= 0.0018).

Since theophylline can effectively suppress the production of IL-13 by macrophages, we then examined whether other cAMP-related agents have the same effects. The designated dosages of two phosphodiesterase inhibitors type IV (etazolate and rolipram) and two cAMP-elevating agents (forskolin and dibutyryl-cAMP) were added to macrophages (PMA-treated THP-1 cells) separately for 24 hours. Dose-dependent suppression of IL-13 mRNA expression were observed in all four drugs that could increase intracellular cAMP levels (α = 0.05, *p *= 0.0009 compared to control) (Figure 5A). Similar results were obtained with ELISA (α = 0.05, *p *= 0.0018 compared to control) (Figure 5B).

### Effects on LTC4 expression

**Figure 6:**
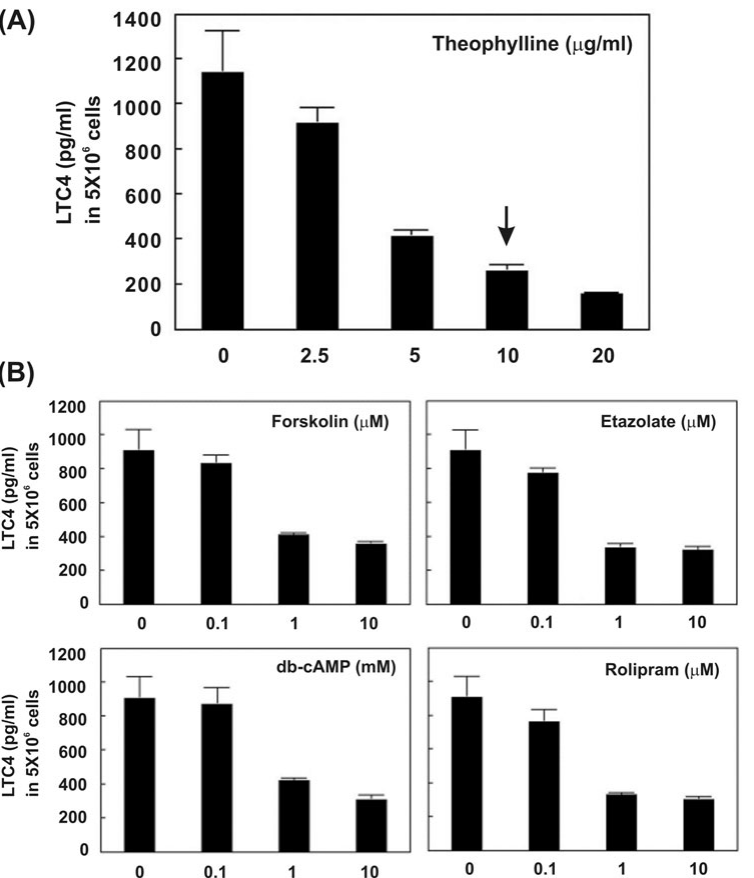
LTC4 secretion by macrophages was suppressed by theophylline and cAMP signaling regulators in a dose-dependent pattern. The cultured medium of macrophages treated with tested drugs was collected to perform ELISA. (A) LTC4 protein secretion was reduced by theophylline stimulation. (B) Etazolate, rolipram, forskolin, and db-cAMP (dibutyryl-cAMP) also suppressed LTC4 protein secretion. Arrow indicates the concentration of theophylline treatment corresponding to the clinical plasma levels (10 mg/L).

The LTC4 is the downstream target of IL-13. Theophylline and other four cAMP-related drugs (etazolate, rolipram, forskolin, and db-cAMP) could dose-dependently suppress LTC4 secretion by macrophages (Figure 6). As shown in Figure 6A, LTC4 production in macrophages (PMA-treated THP-1 cells) was significantly reduced to 78.34%, 34.63%, 23.32%, and 13.51% of the levels seen in the absence of the drug, respectively, with different dosages of theophylline. Similar results were observed in macrophages (PMA-treated THP-1 cells) treated with other cAMP-related drugs (Figure 6B).

## Discussion

Macrophages are key inflammatory cells that have been documented to play a critical role in various airway disorders [8]. In this study, we analyzed the gene expression profiles of macrophages in response to theophylline. A panel of inflammation related genes was identified, as well as genes associated with angiogenesis, cell adhesion, cell motility, signal transduction, and cell proliferation that are dose-dependently down- or up-regulated by theophylline. Our results revealed that 45 genes were up-regulated and 30 genes were down-regulated by theophylline (supplemental Tables 1 and 2). We also found that theophylline can down-regulate IL-13 expression in macrophages through cAMP mediation, which further leads to decreased LTC4 production. Our results provide positive evidence supporting the role of theophylline as a regulator of inflammation.

In this report, interferon regulatory factor 7 (IRF-7) and CD36 were both suppressed by theophylline in macrophages, especially in high dosages (Figures 1 and 2A, and Supplemental Table 2). IRF-7 has been studied extensively in viral infection [27] and can induce the gene expressions of interferon and cytokine [28]. Interestingly, an over-expression of IRF-7 can trigger monocyte differentiation towards macrophages and induce cell cycle arrest, suggesting a different function for IRF-7 in innate immunity [28]. Furthermore, CD36 is a multi-functional receptor that may play important roles in monocyte/macrophage biology, especially in atherogenic and inflammatory processes [29,30].

Airway inflammation in asthma is regulated by a complex network of cytokines. We found that the expressions of several cytokines were altered within the period of theophylline stimulation (Figure 2B and supplemental Tables 1 and 2). Theophylline can suppress IL-5 and IL-13 production by stimulating peripheral blood nuclear cells (PBMC) [31]. Decreased expression of immuno-regulatory cytokines, including IL-12, IL-18, or interferon gamma, can strengthen the inflammatory process and play regulatory roles in asthma by modifying Th2 lymphocyte responses [32]. Using a mouse model of allergic inflammation, it has been shown that GMCSF significantly contributes to the development of allergic airway inflammation, and that dexamethasone can completely inhibit GMCSF release [33]. Our findings reveal similar results in the suppression of *IL-5*, *IL-18*, and *GMCSF *in macrophages with theophylline (Figure 2B).

IL-13 is an immuno-regulatory cytokine secreted predominantly by activated Th2 cells [34], and induces dramatically different patterns of gene expression in primary cultures of airway epithelial cells, airway smooth muscle cells, and lung fibroblasts [35]. IL-13 expression is not only in T cells and mast cells but also in both normal alveolar macrophages and those from subjects with pulmonary fibrosis [36]. Some reports demonstrate that IL-13 is overproduced in asthma and have implicated IL13 in pathogenesis of inflammation and airway remodeling responses [37-39]. Although the contribution of macrophage derived IL-13 to disease is still not clear, it has been considered for therapy target because of its ability to stimulate inflammatory and airway hyperreactivity responses. In this study, there is strong evidence supporting that IL-13 expression is down-regulated by theophylline in a dose-dependent manner (Figures 3 and 4). We also further confirmed the mRNA expression and protein secretion of IL-13 with RTQ-RT-PCR and ELISA.

In macrophages (PMA-treated THP-1 cells), *IL-13Rα1 *mRNA expression was inhibited by theophylline, whereas *IL-13Rα2 *mRNA expression increased (Figure 2B). IL-13 modifies cell behavior by activating the signal transducer and activator of transcription 6 (STAT-6). Consequently, not only IL-13 concentration but also the density of IL-13Rα1 expression may determine the role of IL-13 in the regulation of inflammatory responses in affected tissues. However, not all responses to IL-13 on monocytes and macrophages are dependent on signaling via IL-13Rα1 and significant STAT6 activation [40]. Leukotrienes, the products of lipoxygenases, are thought to be important mediators of IL-13-induced asthma phenotype [41]. LTC4 stimulates eotaxin production by IL-13 treated fibroblasts, thereby indirectly inducing eosinophil sequestration [42]. Recently, some studies demonstrated that the regulation of cAMP level by inhibiting PDE activity appears to be involved in the regulation IL-13 release [43,44]. The type IV PDE inhibitors have the potential to exert an anti-inflammatory effect by inhibiting IL-13 production in lymphocyte and peripheral blood mononuclear cells [43,44].

In this study, we also investigated the influence of cAMP pathway on IL-13 and LTC4 expression in macrophage. We found that etazolate and rolipram, which are PDE type IV inhibitors, can significantly inhibit IL-13 and LTC4 production in mRNA and protein level. Similar suppressions are shown in treatment with PKA activator (forskolin and dibutyryl-cAMP). The results indicate that the inhibition of IL-13 and LTC4 might through cAMP and PKA mediation in macrophage. However, the role of PKA in anti-inflammatory effects through cAMP mediation is less established. Although most of the cAMP exerted its downstream effects though the PKA dependent pathway, some actions of cAMP have been reported to be independent of PKA, including the activation of small GTPase Rap1 [45].

In addition, several lines of evidence support that cAMP may act at transcription, post-transcription, or translation levels. For example, cAMP elevating agents can repress NF-kappaB dependent transcription by a variety of mechanism [46], and NF-kappaB is also known to be involved in the induction of TNF-alpha, IL-3, and IL-13 in human mast cells [47]. Although the mechanism involved in the regulation of cAMP and IL-13 is still unclear, this study suggests that a possible pathway of the suppressive effects of theophylline on IL-13 expression may be through a cAMP mediated regulation.

**Figure 7:**
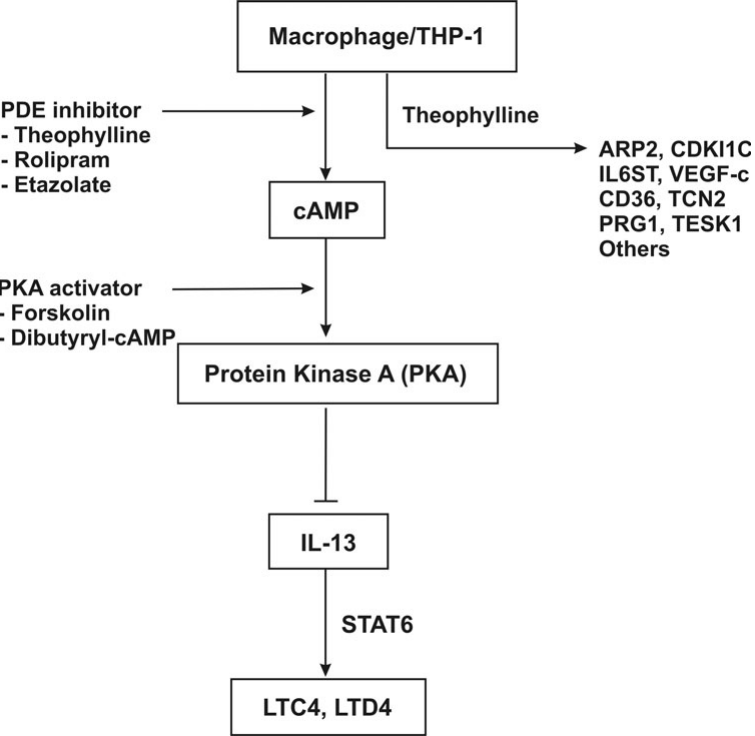
A model for the possible gene regulation in macrophage THP-1 stimulated by theophylline. There are many differentially expressed genes involved in the response to theophylline, such as ARP2, IL6ST, VEGF-c, and IL-13. The suppression of IL-13 by theophylline might be through cAMP pathway and further inhibits the expression of LTC4 and LTD4.

As shown in Figure 7, we summarized a model for the possible gene regulation in macrophages (PMA-treated THP-1 cells) stimulated by theophylline. Our results suggested that the suppression of IL-13 by theophylline may be through the cAMP pathway and further inhibits the expression of LTC4 and LTD4.

## Conclusion

These data may facilitate the understanding of the diverse anti-inflammatory effects of theophylline, as well as the potential contributing role of macrophages in the pathogenesis of asthma. The importance of theophylline as a signal regulator of inflammation should be re-emphasized. Our results suggest that theophylline could down-regulate IL-13 expression in macrophages through cAMP mediation, and further lead to a decrease in LTC4 production, which may have beneficial effects on the therapeutic use of theophylline in pulmonary inflammatory diseases.

## Competing interests

The author(s) declare that they have no competing interests.

## Authors' contributions

PLY performed the RNA isolation, drug treatment and microarray analysis, and drafted the manuscript. MFT performed the alveolar macrophage isolation, culture, drug treatment, ELISA and drafted the manuscript. YCL performed the Northern blotting and real-time RT-PCR experiments. CHW performed the cell culture and real-time RT-PCR experiments. WYL performed the bronchoscopic examination and alveolar macrophage isolation. JJWC and PCY participated in the conception and design of the study as well as proof read the manuscript. All authors read and approved the final manuscript.

## Supplementary Material

Additional File 1 Supplemental Methods: including microarray system, preparation of biotin-labeled cDNA targets, microarray hybridization and colorimetric detection, and image processing and data analysis. Supplemental Table 1. Differential genes up-regulated by theophylline in macrophage THP-1. Supplemental Table 2. Differential genes down-regulated by theophylline in macrophage THP-1. Click here for file
