# Supplementary material for: Global expression profiling of theophylline response genes in macrophages: evidence of airway anti-inflammatory regulation
Source: Respir Res. 2005 Aug 8;6(1):89. doi: 10.1186/1465-9921-6-89 (PMC1215521; doi:10.1186/1465-9921-6-89)
Supplement: Additional File 1 — Supplemental Methods: including microarray system, preparation of biotin-labeled cDNA targets, microarray hybridization and colorimetric detection, and image processing and data analysis. Supplemental Table 1. Differential genes up-regulated by theophylline in macrophage THP-1. Supplemental Table 2. Differential genes down-regulated by theophylline in macrophage THP-1. [file 1465-9921-6-89-S1.pdf]

Data Supplement

Global expression profiling of theophylline response genes in macrophages: evidence of airway anti-inflammatory regulation

Pei-Li Yao[†1,2], Meng-Feng Tsai[†1,2], Yi-Chen Lin[1,2], Chien-Hsun Wang[2,3], Wei-Yu Liao[1], Jeremy J.W. Chen*[2,3] and Pan-Chyr Yang*[1,2]

Address: [1]Department of Internal Medicine, National Taiwan University Hospital, No. 7, Chung-Shan South Rd., Taipei 100, Taiwan, [2]NTU Center for Genomic Medicine, National Taiwan University College of Medicine, Taipei 100, Taiwan and [3]Institutes of Biomedical Sciences and Molecular Biology, National Chung-Hsing University, No. 250, Kuo-Kuang Rd., Taichung 40227, Taiwan

Email: Pei-Li Yao - dalen@mail.utexas.edu; Meng-Feng Tsai - tsai@microarray.mc.ntu.edu.tw; Yi-Chen Lin - vance@microarray.mc.ntu.edu.tw; Chien-Hsun Wang - topo@cm1.hinet.net; Wei-Yu Liao - daphyu@ha.mc.ntu.edu.tw; Jeremy J.W. Chen* - jwchen@dragon.nchu.edu.tw; Pan-Chyr Yang* - pcyang@ha.mc.ntu.edu.tw

* Corresponding authors; [†] Equal contributors

Correspondence to: Pan-Chyr Yang, Department of Internal Medicine, National Taiwan University Hospital, No. 7, Chung-Shan South Rd., Taipei, 100, Taiwan. Phone: +886-2-2356-2905; Fax: +886-2-2358-2867; E-mail: pcyang@ha.mc.ntu.edu.tw

Supplemental methods

Microarray system

Human EST clones with putative gene names were obtained from the IMAGE consortium libraries through its distributor (Research Genetics, Huntsville, AL) (1). These gene clones were derived from various tissues and in different library constructs. Most of the clones have been partially sequenced, and the sequence information is available as expressed sequence tags (ESTs) from dbEST of GeneBank (2). The 9,600 PCR-amplified cDNA in V-bottomed 96-well microtiter plates were distributed onto a positively charged nylon membrane (measured 18 mm by 27 mm) with spots spaced 200 μm apart by an arraying machine (Wittech, Taipei, Taiwan), with position resolution and repeatability better than ± 5 μm.

Preparation of biotin-labeled cDNA targets

Total RNA of macrophage was extracted using RNAzolB (Tel-Test, Friendswood, TX). The mRNA was purified from total RNA using an mRNA Isolation Kit (Qiagen, Hilden, Germany), following the manufacturers' protocol, and five micrograms of mRNAs were used for preparation of the cDNA target. The labeling reactions were performed during reverse transcription in the presence of 6 μM random primers; 0.5 mM each dATP, dCTP,

and dGTP; 40 μM dTTP; 40 μM biotin-16-dUTP (Roche Molecular Biochemicals; Mannheim, Germany); 1X reaction buffer; 10 mM DTT; 0.5 unit/μl Ribonuclease inhibitor (Invitrogen, Gaithersburg, MD); and 200 units of MMLV reverse transcriptase (Invitrogen) in a 50-μl solution. The reaction mixture was incubated at room temperature for 10 minutes, then transferred to 42 °C for 90 minutes, and was stopped by heating the reaction mixture to 99 °C for 5 minutes. The RNA was degraded by adding 5.5 μl of 3 M NaOH followed by a 30-min incubation at 50 °C. The labeled samples were neutralized by the addition of 5.5 μl of 3 M acetic acid, and then precipitated by adding 50 μl of 7.5 M ammonium acetate, 20 μg of linear polyacrylamide as a carrier, 375 μl of absolute alcohol, and water, to make a total of 525 μl. The solution was mixed evenly and stood at –80 °C for 30 min, then was centrifuged at 14,000 rpm for 20 min to precipitate a single stranded DNA target. The pellet was washed with 1 ml of 75% of ethanol and dried by speed vacuum, then dissolved in 5 μl of de-ionized water.

Microarray hybridization and Colorimetric detection

The membrane carrying the double-stranded cDNA probes was pre-hybridized in 5 ml of hybridization buffer (5× SSC, 0.1% N-lauroylsarcosine, 0.1% SDS, 1% blocking reagent made by Roche Molecular Biochemicals, and 50 μg/ml salmon sperm DNA) at

68°C for 1 hour before hybridization was carried out. cDNA targets were resuspended in 100 μl hybridization buffer containing 200 μg/ml $d(A)_{10}$ and 400 μg/ml human COT-1 DNA (Invitrogen) to prevent non-specific binding, and were hybridized to the cDNA fragments on the membrane by a Southern hybridization procedure. The 100 μl reaction mixture was sealed with the microarray membrane in a hybridization assembly (SureSeal, Hybaid, Middlesex, UK) at 68°C for 12 hours. The membrane was then washed with 2× SSC containing 0.1% SDS for 5 min at room temperature followed by three washes with 0.1× SSC containing 0.1% SDS at 65 °C for 15 min each (3-5).

After hybridization, the membrane was blocked by 1 ml of 1% blocking reagent (Roche) containing 2% dextran sulphate at room temperature for 1 hour, and then was rinsed with 1 X TBS buffer solution (10 mM Tris-HCl, pH 7.4, 150 mM NaCl, 0.3% BSA). To detect the spots on the membrane in a single-color mode, β-galactosidase-conjugated streptavidin (Invitrogen) was employed. The membrane was incubated with a 3-ml mixture containing 700 X diluted Strep-Gal (1.38 units/ml, enzyme activity), 4% polyethylene glycol 8000 (Sigma, St. Louis, MO), and 0.3% BSA in 1 X TBS buffer for 2 hours. The membrane was then washed with 1 X TBS buffer three times for 10 minutes each. The chromogen was generated by treating the membrane with X-gal substrate containing 1.2 mM X-gal, 1 mM $MgCl_2$, 3 mM $K_3Fe(CN)_6$, and 3 mM $K_4Fe(CN)_6$ in 1 X

TBS buffer for 1 hour at 37 °C for the β-galactosidase reaction. The color development reactions were stopped by 1 X PBS containing 20 mM EDTA. After color development, the cDNA molecules labeled with biotin yielded a blue chromogen. To measure the expression levels of the genes, the microarray was scanned using a flatbed scanner (PowerLook 3000, UMAX, Taipei, Taiwan).

Image processing and Data analysis

The images captured by a scanner were converted from true color to a gray scale, and digitized by analysis software GenePix 3.0 (Axon, Union City, CA). To reduce the variation arising between microarray experiments, the intensity values of the spots were rescaled using global normalization method based on the sum of all spots' intensities in a microarray (6). Only signal values of spots above 3,000 were considered meaningful in the membrane format microarray system (*i.e.* the intensity of a spot less than 3,000 was hardly distinguished from the background, so an intensity value below 3,000 was given the value of 3,000). To focus on genes that were significantly regulated under studied conditions, genes with a greater than two-fold change across all dosage points were selected and normalized by a mean center to render the mean value equal to zero and the magnitude equal to one (sum of the squares of the values) by free software, Cluster

(Stanford University and Massachusetts Institute of Technology) (7). Those selected genes were clustered into 16 groups by SOMs (self organization maps) algorithm software, GeneCluster 1.1 (Stanford University and Massachusctts Institute of Technology) (8). The selected genes were clustered using a hierarchical cluster method (TreeView, Stanford University and Massachusetts Institute of Technology).

References

1. Lennon G, Auffray C, Polymeropoulos M, Soares MB: **The I.M.A.G.E. Consortium: An integrated molecular analysis of genomes and their expression.** *Genomics* 1996, **33:**151-152.
2. Boguski MS, Tolstoshev CM, Bassett DE: **Gene discovery in db EST.** *Science* 1994, **265:**1993-1994.
3. Chen JJ, Wu R, Yang PC, Huang JY, Sher YP, Han MH, Kao WC, Lee PJ, Chiu TF, Chang F, Chu YW, Wu CW, Peck K: **Profiling expression patterns and isolating differentially expressed genes by cDNA microarray system with colorimetry detection.** *Genomics* 1998, **51:**313-324.
4. Hong TM, Yang PC, Peck K, Chen JJ, Yang SC, Chen YC, Wu CW: **Profiling the down stream genes of tumor suppressor PTEN in lung cancer cells by cDNA microarray.** *Am J Respir Cell Mol Biol* 2000, **23:**355-363.
5. Chen JJ, Peck K, Hong TM, Yang SC, Sher YP, Shih JY, Wu R, Cheng JL, Roffler SR, Wu CW, Yang PC: **Global analysis of gene expression in invasion by a lung cancer model.** *Cancer Res* 2001, **61:**5223-5230.
6. Ball CA, Chen Y, Panavally S, Sherlock G, Speed T, Spellman PT, Yang YH: **An introduction to microarray bioinformatics.** In *DNA Microarray: A Molecular*

Cloning Manual. Edited by Bowtell D and Sambrook J. New York: Cold Spring Harbor; 2002:536-543.

7. Iyer VR, Eisen MB, Ross DT, Schuler G, Moore T, Lee JC, Trent JM, Staudt LM, Hudson J Jr. Boguski MS, Lashkari D, Shalon D. Botstein D, Brown PO: **The transcriptional program in the response of human fibroblasts to serum.** *Science* 1999, **75:**83-87.

8. Tamayo P, Slonim D, Mesirov J, Zhu Q, Kitareewan S, Dmitrovsky E, Lander ES, Golub TR: **Interpreting patterns of gene expression with self-organizing maps: methods and application to hematopoietic differentiation.** *Proc Natl Acad Sci USA* 1999, **96:**2907-2912.

Supplemental Table 1. Differential genes up-regulated by theophylline in macrophage THP-1.

Abbreviation	Gene Name	Accession No.	Relative Expression Ratio: Concentration of Theophylline (increasing)				
GPM6A	Glycoprotein M6A	NM_005277	1.00	3.22	4.27	2.91	5.50
NUTF2	Nuclear transport factor 2	NM_005796	1.00	2.70	5.09	4.00	5.22
PC5	Prohormone convertase 5 precursor	NM_006200	1.00	2.40	3.52	2.03	4.56
BLNK	B cell linker protein	NM_013314	1.00	1.63	3.77	2.48	4.21
eIF4B	Eukaryotic translation initiation factor 4B	NM_001417	1.00	2.25	4.46	4.25	4.08
GFPT1	Glutamine-fructose-6-phosphate transaminase 1	NM_002056	1.00	1.60	3.02	1.37	4.06
STAT3	Signal transducer and activator of transcription 3	NM_003150	1.00	2.18	3.13	2.76	3.79
ILF1	Interleukin enhancer binding factor 1	NM_004514	1.00	1.59	3.02	2.05	3.72
UBQLN2	Ubiquilin 2	NM_013444	1.00	2.20	2.89	1.54	3.71
LPL	Lipoprotein lipase	NM_000237	1.00	2.28	2.98	1.96	3.43
GMRP1	K^+ channel tetramerization protein	AY305862	1.00	1.55	1.56	1.50	3.20

SDS	Serine dehydratase	NM_006843	1.00	2.01	3.01	1.76	2.96
RGS16	Regulator of G-protein signaling 16	NM_002928	1.00	1.86	2.46	2.04	2.93
TESK1	Testis-specific kinase 1	NM_006285	1.00	1.03	2.02	1.99	2.80
TGIF	TGFB-induced factor	NM_003244	1.00	1.64	1.69	1.31	2.45
NEDD4L	Ubiquitin-protein ligase NEDD4-like	NM_015277	1.00	1.61	1.82	1.33	2.57
VEGFC	Vascular endothelial growth factor C	NM_005429	1.00	2.12	2.83	1.69	2.41
DSCR1	Down syndrome candidate region 1	NM_004414	1.00	1.36	1.65	1.09	2.37
BCR	Breakpoint cluster region protein	NM_004327	1.00	1.10	1.90	1.69	2.32
TUSC3	Tumor suppressor candidate 3	NM_006765	1.00	2.82	3.88	3.35	2.05
OSBP	Oxysterol binding protein	NM_002556	1.00	1.03	1.48	1.20	2.05
HSPA2	Heat-shock 70kD protein-2	NM_021979	1.00	1.28	3.33	2.97	1.75
NRXN3	Neurexin III	NM_004796	1.00	1.45	3.56	2.02	1.72
ITGB2	Integrin, beta 2	NM_000211	1.00	1.77	2.00	1.38	1.70
IFNB2	Interferon-beta-2	NM_000600	1.00	1.30	1.19	2.00	1.69
LST1	Leukocyte-specific transcript 1	NM_007161	1.00	1.98	3.79	3.35	1.64

PLD3	Phospholipase D3	NM_012268	1.00	1.63	2.00	1.32	1.63
CAV1	Caveolin 1	NM_001753	1.00	1.36	1.45	2.35	1.60
IRS1	Insulin receptor substrate 1	NM_005544	1.00	1.53	2.00	1.46	1.57
eIF3S10	Eukaryotic translation initiation factor 3, subunit 10 theta, 150/170kDa	NM_003750	1.00	1.18	1.35	2.00	1.56
eIF2S3	Eukaryotic translation initiation factor 2, subunit 3, gamma	NM_001415	1.00	1.00	0.30	1.47	2.00
IL6ST	Interleukin 6 signal transducer (gp130, oncostatin M receptor)	NM_002184	1.00	1.00	1.30	1.00	2.00
IHH	Indian hedgehog (Drosophila) homolog	NM_005403	1.00	0.22	1.40	2.00	1.46
TCN2	Transcobalamin II	NM_000355	1.00	1.40	2.00	2.00	1.43
CTSB	Cathepsin B	NM_001908	1.00	1.26	2.00	1.24	1.41
TPMT	Thiopurine methyltransferase	NM_000367	1.00	1.45	2.00	1.45	1.40
CDKI1C	Cyclin-dependent kinase inhibitor 1C	NM_000076	1.00	1.47	2.00	1.48	1.38
EVI1	Ecotropic viral integration site 1	NM_005241	1.00	1.25	2.00	1.55	1.35

PRG1	Platelet proteoglycan 1	NM_002727	1.00	1.04	1.04	2.00	1.34
MAP2	microtubule-associated protein 2	NM_002374	1.00	1.47	2.72	1.66	1.30
FKBP38	FK506 binding protein 8	NM_012181	1.00	0.91	1.26	2.00	1.30
DOCK1	Dedicator of cytokinesis 1	NM_001380	1.00	2.70	2.12	2.74	1.26
ACTN3	Actinin, alpha 3	NM_001104	1.00	1.93	1.20	2.00	1.24
SYPL	Synaptophysin-like protein, Pantophysin	NM_006754	1.00	1.46	1.30	2.00	1.21
CD3G	CD3G antigen, gamma polypeptide	NM_000073	1.00	1.00	1.00	2.24	1.07

Supplemental Table 2. Differential genes down-regulated by theophylline in macrophage THP-1.

Abbreviation	Gene Name	Accession No.	Relative Expression Ratio<br>Concentration of Theophylline				
ABL2	v-abl Abelson murine leukemia viral oncogene homolog 2	NM_005158	1.00	0.72	0.42	0.60	0.25
PRKCA	Protein kinase C, alpha	NM_002737	1.00	0.73	0.84	0.79	0.29
TRAF1	TNF receptor-associated factor 1	NM_005658	1.00	0.55	0.44	0.52	0.29
UGT2B15	UDP glucuronosyltransferase precursor	NM_001076	1.00	0.80	0.87	0.88	0.30
PPP3CB	Phosphatase 3, catalytic subunit, beta isoform	NM_021132	1.00	0.62	0.72	0.63	0.31
PTP4A1	Tyrosine phosphatase type IVA, member 1	NM_003463	1.00	0.78	0.67	0.76	0.32
eIF5	Eukaryotic translation initiation factor 5	NM_001969	1.00	0.57	0.60	0.46	0.34
TAFII55	TFIID subunit TAFII55	NM_005642	1.00	0.77	0.79	0.52	0.37

CAPZA1	Capping protein alpha	NM_006135	1.00	0.76	0.72	0.70	0.39
SUI1	Translation factor sui1	NM_005875	1.00	0.84	0.99	0.84	0.39
GSTM2	Glutathione S-Transferase M2	NM_000848	1.00	0.94	0.94	0.60	0.42
CD36	CD36 antigen	NT_000027	1.00	0.73	0.82	0.71	0.42
SUPT6H	Suppressor of Ty (S.cerevisiae) 6 homolog	NM_010799	1.00	0.83	0.80	0.71	0.43
EVI2B	Ecotropic viral integration site 2B	NM_006495	1.00	0.75	0.75	0.72	0.43
IL-13	Interleukin-13	NM_002188	1.00	0.91	0.77	0.50	0.47
GNB1	Guanine nucleotide binding protein, beta polypep 1	NM_002074	1.00	0.67	0.72	0.87	0.48
ALDOC	Aldolase C	NM_005165	1.00	0.95	0.80	0.83	0.48
eIF3S6	Eukaryotic translation initiation factor 3, subunit 6	NM_001568	1.00	0.85	0.76	0.66	0.48
ARP2	Actin-related protein 2	NM_005722	1.00	0.89	0.96	0.86	0.50
IGFBP5	Insulin-like growth factor binding Protein 5	NM_000599	1.00	0.93	0.94	0.79	0.50
RAD9	RAD9 (S. pombe) homolog	NM_004584	1.00	0.94	0.97	0.91	0.50
ARP3	Actin-related protein 3	NM_005721	1.00	0.76	0.77	0.74	0.50

CTPS	CTP synthase	NM_001905	1.00	0.75	0.88	0.80	0.50
SudD	Suppressor of bimD6	NG_002288	1.00	0.85	0.83	0.82	0.50
HADHSC	L-3-hydroxyacyl-CoA dehydrogenase	NM_005327	1.00	0.91	0.77	0.87	0.50
SSR1	Signal sequence receptor, alpha	NM_003144	1.00	0.82	0.86	0.86	0.50
PPP2CA	Protein phosphatase 2 (formerly 2A), catalytic subunit, alpha isoform	NM_002715	1.00	0.90	0.92	0.82	0.50
IRF7	Interferon regulatory factor 7	NM_001572	1.00	0.97	0.93	0.95	0.50
MUC5B	Mucin 5, subtype B	NT_009237	1.00	0.86	0.95	0.93	0.50
PEA15	Phosphoprotein enriched in astrocytes 15	NM_003768	1.00	0.92	0.94	0.94	0.50
